# Redesign of the Hannover Coupler: Optimized Vibration Transfer from Floating Mass Transducer to Round Window

**DOI:** 10.1155/2018/3701954

**Published:** 2018-05-14

**Authors:** Mathias Müller, Rolf Salcher, Nils Prenzler, Thomas Lenarz, Hannes Maier

**Affiliations:** Cluster of Excellence Hearing4all, Department of Otolaryngology and Institute of Audioneurotechnology (VIANNA), Hannover Medical School, Hannover, Germany

## Abstract

**Introduction:**

In order to reduce the large variations in clinical outcomes of patients with implanted MED-EL Floating Mass Transducer (FMT) at the round window (RW), several approaches were proposed to optimize FMT-RW coupling. Our previous study showed improved FMT-RW coupling by applying static RW loads utilizing the “Hannover Coupler” (HC) FMT-prosthesis but also demonstrated insufficient low frequency performance. Hence, a redesigned HC version (HCv2) was investigated in this study.

**Methods:**

Experiments were performed in ASTM F2504-05 compliant fresh human temporal bones. The HCv2 is a FMT-prosthesis redesigned from a previous prototype to specifically improve low frequency performance. Stapes footplate (SFP) displacements in response to acoustic stimulation of the tympanic membrane and to FMT-RW stimulation at varying static force (0–100 mN) were measured by Laser-Doppler vibrometry.

**Results:**

SFP displacements were highly dependent on the applied RW load and had a global maximum at 15 mN when averaged at speech relevant frequencies (0.5–4 kHz). SFP responses at frequencies ≤ 1 kHz were up to 25 dB higher than responses achieved with the previous HC version.

**Conclusion:**

Optimizing the HC prosthesis design resulted in improved SFP responses to RW stimulation especially at lower frequencies (≤1 kHz).

## 1. Introduction

The MED-EL Vibrant Soundbridge® (VSB) is an active middle ear implant (AMEI) with a Floating Mass Transducer (FMT) that is typically attached to the long process of the incus of the ossicular chain. Similar to hearing aids it provides amplification, but in contrast it stimulates the ossicles mechanically instead of using a sound output to the tympanic membrane. During the last 20 years it has become a well-established treatment for patients suffering from sensorineural hearing loss. In 2005 Colletti et al. [1] successfully implanted the VSB at the cochlear round window (RW), demonstrating that circumventing and substituting the middle ear with an AMEI is feasible (Figure 7, supplementary information). Since then the application of the VSB has been extended from pure sensorineural to pathologies with dysfunctional middle ears, that is, conductive and mixed hearing losses. However, placement of the FMT at the round window membrane (RWM) remains surgically challenging [2] and RW stimulation is still subject to large variations in clinical outcomes [3, 4].

A geometric mismatch in diameter between the FMT (diameter ~ 1.8 mm) and the RWM (diameter = 1.5–1.9 mm [5]) can be a limiting factor in establishing optimal coupling. In recent years several approaches addressing the coupling efficiency were proposed, mainly involving interposing tissue or artificial materials [6], bracing the FMT to the hypotympanum [7], or the usage of commercially available RW couplers [8]. The most common commercially available RW couplers are the hemispherical titanium RW-coupler or, more recently, the conically shaped silicone-based soft coupler [9]. Although these approaches potentially improve coupling between FMT and RWM the occurring static loads of the actuator to the RW membrane are typically undefined. Consequently, the coupling force between RWM and FMT is unknown.

In a previous study of our group [10] we could demonstrate the feasibility of a novel prosthesis for the FMT termed “Hannover Coupler” (HC) that allows for RW stimulation at controlled static RW preloads. Stimulation with the HC showed improved stapes footplate (SFP) displacement at frequencies ≥ 1 kHz at an optimal RW load of 4 mN. However, results fell below optimal SFP responses reported in literature at frequencies < 1 kHz.

In this work we systematically investigated the performance of a redesigned and optimized version of the HC termed “Hannover Coupler v2” (HCv2) and its influence on RW stimulation by applying well defined static forces to the RWM (0–100 mN).

## 2. Methods

### 2.1. Design of the Hannover Coupler v2

**Figure 1:**
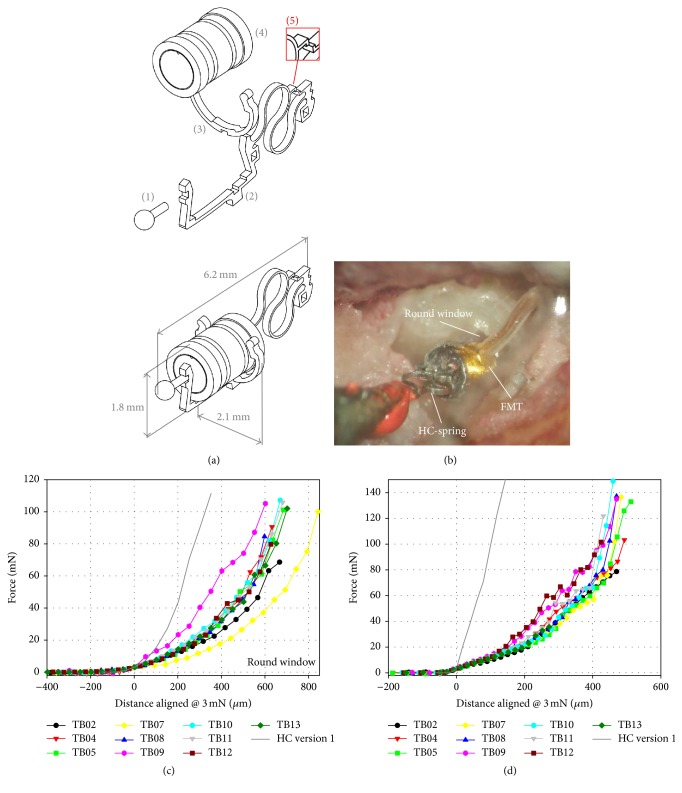
(a) The upper panel shows the exploded view of the HCv2 assembly consisting of tip (1), frame with spring (2), clip (3), the FMT (4), and a visual indicator to estimate the applied static force (5). The lower panel shows the assembled HCv2 with the FMT. (b) The HCv2/FMT assembly positioned at the RW membrane. The free end of the spring was fixed with sealing wax to a holder. (c) Static forces versus displacement when the HCv2/FMT was displaced against the RW membrane in temporal bone experiments. (d) Force-displacement characteristic of the HCv2/FMT alone, obtained by displacing it against an incompliant target. Forces were aligned at a threshold of 3 mN. The gray solid lines in (c) and (d) display an example measurement with the previous HC prototype [10].

The HCv2 is a supplementary, light-weight prosthesis (*m* = 2.4 mg) for the FMT that consists of three titanium components: frame, tip, and clip (Figure 1(a)). Its layout [11] is based on the preceding HC prosthesis design [10]. Different from the preceding prototype, the HCv2 spring is now part of the prosthesis frame to increase robustness. All three components of the HCv2 are laser-welded into a single piece that can be clipped to the FMT body (Figure 1(a)). Hence, the FMT's radial and axial fixation is ensured by the prosthesis clip and frame.

Like in the previous HC version [10] the prosthesis tip is spherical with a diameter (*d* = 0.5 mm) that is well below the typical diameter of the RW membrane [5]. The spring at the prosthesis back, however, is a redesign of the previous HC prototype. In contrast to the previous prototype it has a pronounced S-shape, allowing for a uniform deformation over a wide range of axial compression. The layout was derived by finite element simulation under the premise of enhancing low frequency (≤1 kHz) performance of the prosthesis by implementing a softer spring characteristic and to simplify intraoperative placement of the device. In surgical applications the HCv2/FMT assembly is supposed to be placed in the enlarged RW niche with the spring's rear end in contact with the bony wall opposite to the RW membrane. Two horizontal metal bars at the spring's back end (Part (5) of Figure 1(a)) serve as a visual indicator of the applied static preload to the RW membrane. The two bars coincide when a static load of ~20 mN is reached.

### 2.2. Human Cadaver Temporal Bone Preparation and Setup

Fresh human cadaver temporal bones (TBs) used in the experiments were prepared by experienced surgeons (Rolf Salcher, Nils Prenzler). Details of the TB preparation and the measurement setup are described in an earlier publication [10].

In short, stimulation of the TBs was performed either acoustically via the external auditory canal and tympanic membrane by a loudspeaker (DT-48, Beyer Dynamic, Germany) or mechanically by the HCv2/FMT placed at the RW membrane. The loudspeaker (LS) or actuator was driven by a custom written multisine signal with equal amplitudes (LS input approx. −27 dB re 1 *V*_RMS_, FMT input approx. −44 dB re 1 *V*_RMS_) at 0.125, 0.25, 0.5, 1.0, 2.0, 3.0, 4.0, 6.0, 8.0, and 10.0 kHz. In addition, the FMT was driven by single sine wave signals at 0.5, 1, 1.5, and 2 kHz (input voltage = 100 mV_pp_) to measure the total harmonic distortion (THD). Only higher harmonics ≤ 10 kHz with a signal-to-noise ratio (SNR) ≥ 12 dB were included in the determination of the THD. Similarly, in all other measurements (e.g., SFP displacement amplitudes) data was only included in the analysis if it exceeded a SNR of 12 dB.

Displacement amplitudes of the stapes footplate (SFP) and HCv2/FMT were measured using a Laser-Doppler Velocimeter (LDV) with a micromanipulator (OFV-5000, HLV-MM30, Polytec, Germany) on a surgical microscope (OPMI-1, Zeiss, Germany). Small pieces (~0.04 mm^2^) of reflective tape were placed at the SFP and the backside of the HCv2 spherical tip to enhance reflected signal intensity.

It needs to be mentioned that LDV measurements of the SFP are not the optimal method to determine equivalent sound pressure output level in reverse direction stimulation of the cochlea [12]. Here, the measurement of intracochlear pressure differences would be more adequate but would have required openings to the scala vestibuli and scala tympani. The application of large forces (~100 mN) to the RW as done here is associated with pronounced volume displacements. This in return would have required preventive measures to avoid leakage at the openings for the pressure probes. Hence, we decided to use LDV on the stapes footplate leaving the cochlear closed to determine relative stimulation efficiency.

### 2.3. Hannover Coupler v2 Setup and Positioning

In experiments the distal end of the HCv2 spring was rigidly connected to the conical tip of a metal rod by a drop of sealing wax. The metal rod was fixed to a force sensor (LSB 210, Futek Advanced Sensor Technology, USA) and mounted on a three-axis micromanipulator (MM3301, WPI, Germany). The setup was located on a vibration isolated table (LW3048B, Newport, Germany). The HCv2/FMT assembly was positioned perpendicular to the RWM with the tip ~100 *μ*m away from the membrane (Figure 1(b)). It was verified that the tip had no visible contact to the RWM and the bony surroundings. At this initial position the force sensor was reset to zero.

After this initialization, the HCv2/FMT was displaced in increments of 50 *μ*m towards the RWM, and the force readings were documented both directly after applying a step and after performing LDV measurements (relaxation time of ~10 min). Displacement steps were repeated until a static RW load of 100 mN was exceeded or the bone contact hindered further advancement. At each 50 *μ*m step SFP and HCv2 tip displacement amplitudes in response to acoustic or FMT stimulation were measured by the LDV.

### 2.4. Averaging Procedure

The force sensor was set to zero prior to initial contact between HCv2 tip and RWM where no physical contact was visible. That gave access to a defined initial force but the corresponding distance was arbitrary in each experiment. Hence, individual force-displacement curves from TB experiments lacked a shared distance reference point. In accordance with previous publications [10, 13] force-displacement curves were linearly interpolated (increment = 1 *μ*m), and distances were then shifted until a distance of 0 *μ*m corresponded to a selected force threshold of 3 mN.

The initial static force was determined at the 50 *μ*m step before the first positive force indication was registered. When averaged between experiments this initial force was *F*_mean_ = −0.31 mN. Most likely, the negative value derives from a liquid meniscus between RWM and HCv2 tip that attracted the tip towards the RWM. Data from LDV measurements were linearly interpolated between adjacent displacement steps and then averaged between TB experiments at the following RW loads: −0.31, 2, 4, 6, 8, 10, 15, 20, 30, 40, 50, 60, 70, 80, 90, and 100 mN.

## 3. Results

### 3.1. Stapes Footplate Responses to Acoustic Stimulation (No RW Load)

Out of 13 tested temporal bones 10 fulfilled the extended ASTM acceptance criteria as described by Rosowski et al. [14] and were included in the analysis. The SFP displacement amplitudes in response to acoustic stimulation of the tympanic membrane (~94 dB SPL) are depicted in Figure 6 (supplementary information).

### 3.2. Static Force-Displacement Characteristics of the HCv2

Figure 1(c) shows the measured aligned force-displacement curves when the HCv2 was moved against the RWM. A static load of 100 mN was reached in 6 out of 10 TBs at a mean distance of 691 ± 81 *μ*m. In the remaining 4 experiments the maximum achievable force was limited by contact to the surrounding bone.

The HCv2 assembly consisting of HCv2 + FMT in parallel to FMT connector cable + force sensor was displaced against an incompliant metal target in order to measure the force-displacement characteristic (Figure 1(d)). Up to a static load of ~20 mN individual curves were similar (standard deviation ≤ 3 mN) but differed from each other at higher static loads (standard deviation 9–11 mN). Assuming a linear range of the HCv2 force-displacement characteristic (Figure 1(d)) at distances 0 to 200 *μ*m an average spring constant of 93.6 N/m was assessed.

### 3.3. SFP Responses to Acoustic Stimulation with RW Load

**Figure 2:**
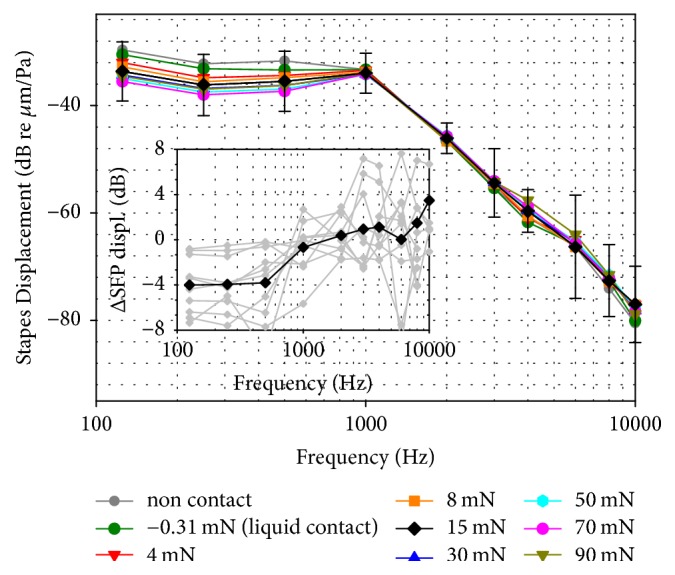
Mean (*n* = 10) SFP displacement amplitudes in response to acoustic stimulation of the tympanic membrane (~94 dB SPL) while the RW was loaded at different static forces (colored lines) and prior to HC placement (gray line). For an RW load of 15 mN the standard deviation is shown by error bars. The inset shows mean (black diamonds) differences of SFP displacements at 15 mN RW load compared to the unloaded state as well as the individual results (gray lines). Negative values correspond to decreased SFP responses compared to the noncontact reference.

Figure 2 depicts the averaged (*n* = 10) SFP responses to acoustic stimulation of the tympanic membrane (~94 dB SPL) with the HCv2 pushed against the RW membrane at different static forces. At frequencies ≤ 1 kHz SFP displacement amplitudes were highest (−30 to −33 dB re *μ*m/Pa) at lowest RW loads (−0.31 mN) and decreased by ~4 dB with increasing static force. SFP responses to acoustic stimulation at higher frequencies (2–10 kHz) showed only a minor dependence on the applied static RW load with increases ≤ 4 dB even at highest forces.

### 3.4. SFP Responses to RW Stimulation

**Figure 3:**
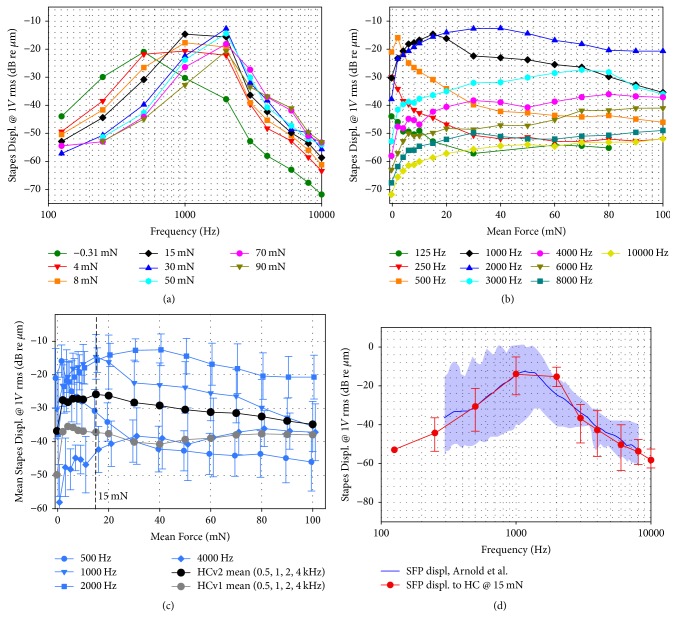
Mean (*n* = 10) SFP displacement amplitudes in response to RW stimulation by the HCv2/FMT at nominal 1 *V*_RMS_ FMT input: (a) as a function of frequency and (b) as a function of applied static RW load. (c) Average SFP displacement amplitudes in response to RW stimulation of selected frequencies from (b) (blue) with SD (error bars). Gross averages for these speech relevant frequencies (0.5, 1, 2, and 4 kHz) are shown for the previous design (gray circles, *n* = 7) and the new HCv2 (black circles, *n* = 10). (d) Mean (*n* = 10) SFP displacement response amplitudes (red line) with min–max variations (error bars, except 125 Hz where only one value with sufficient SNR was available) in response to RW stimulation by the HCv2/FMT at 15 mN static RW load. For comparison SFP responses to RW stimulation from a study of Arnold et al. [6] are displayed as a blue line and corresponding min–max variations are indicated by the blue-shaded area.

Figures 3(a) and 3(b) depict the averaged (*n* = 10) SFP responses to RW stimulation using the HCv2/FMT under varied static RW loads. With increasing static RW load the SFP responses to FMT stimulation at frequencies ≥ 1 kHz increased by 16–25 dB. At low frequencies between 125 and 500 Hz SFP displacement amplitudes decreased by up to 25 dB with increasing RW load.

To assess the optimal static RW load producing highest SFP responses, the SFP displacement amplitudes were averaged at speech relevant frequencies (0.5, 1, 2, and 4 kHz, Figure 3(c)). A global maximum of SFP displacement amplitudes was found at a static RW load of 15 mN.

### 3.5. Transfer Function between FMT and SFP

**Figure 4:**
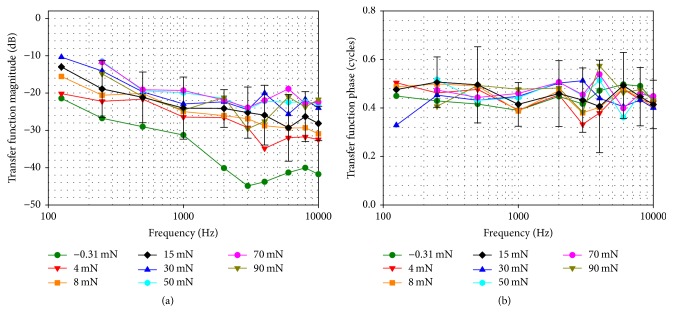
(a) Magnitudes and (b) phases of the mean (*n* = 10) transfer function between HCv2-tip at the RW and SFP at different static RW loads. For an RW load of 15 mN the standard deviations are shown by error bars (except 125 Hz where only one value with sufficient SNR was available).

Figure 4(a) depicts the mean (*n* = 10) transfer function magnitude between the HCv2-tip and the SFP at different static forces. Transfer function magnitudes were lowest (−21 to −45 dB) at the lowest RW load of −0.31 mN which represented liquid contact of HCv2-tip and RW membrane. At higher RW loads transfer function magnitudes increased by 22 dB and saturated above ~30 mN.

The average (*n* = 10) phase (Figure 4(b)) was independent of the applied RW force and was ~0.45 cycles across the measured frequency range (0.125–10 kHz).

### 3.6. Total Harmonic Distortions

**Figure 5:**
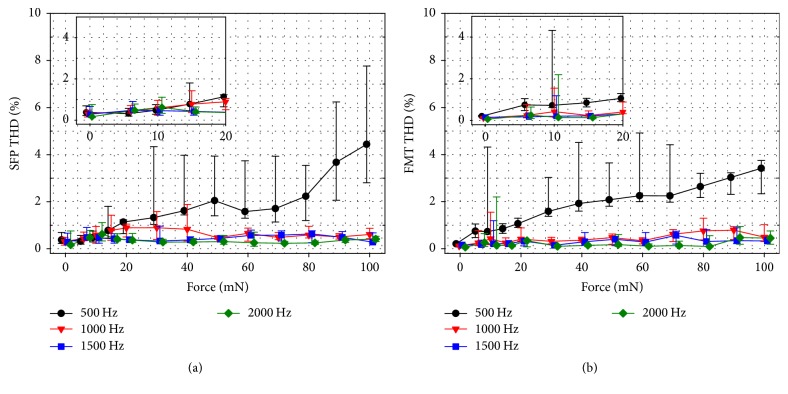
Median (*n* = 10) of total harmonic distortions of (a) SFP and (b) FMT measured at the backside of the HCv2 spherical tip close to the RWM at different static RW loads. Error bars indicate the quartile range. The insets show a magnification of the static force range ≤ 20 mN.

In Figure 5 the total harmonic distortions of (a) the SFP and (b) the FMT at 0.5, 1, 1.5, and 2 kHz as a function of the applied force are shown. Highest average THDs were observed for 500 Hz in both SFP and FMT measurements and reached up to 3–5% at highest RW loads. Average THDs of higher frequencies (1-2 kHz) were ≤1% and showed no pronounced dependence on the static RW load.

## 4. Discussion

Stimulation of the cochlear RW with the FMT is a treatment option for patients with conductive or mixed hearing loss, but clinical outcomes still suffer from large variations [4]. During implantation, applied loads of the FMT to the RW are usually unknown and may contribute to the broad range of results. In this study we investigated the performance and the influence of static RW preload on coupling efficacy with an accessory prosthesis for the FMT termed “Hannover Coupler v2.” The HCv2 was an optimized redesign of a previous coupler version [10]. The novel S-shaped spring was redesigned to standardize contact forces and to improve low frequency (<1 kHz) transmission of the FMT to the RW. In the performed temporal bone experiments no RW rupture was observed when loading the RW membrane with the HC even up to ~100 mN. Comparison with published rupture limits by Ishii et al. [15] results in hypothetical rupture limits (>850 *μ*m) for a similar geometry (*d*_HC-tip_ = 500 *μ*m, *d*_RWM_ = 1.2 mm) which is above the maximum reached in our experiments. However, our experience showed that leaving sufficient bone of the RW niche is crucial for avoiding detachment of the RWM at the borders.

Measured SFP displacement amplitudes showed a strong dependence on the applied static RW load by the HCv2 and increased 16–25 dB at frequencies ≥ 1 kHz (Figure 3). This is consistent with the dependence of SFP responses on RW loading with the previous HC version which showed similar increases (~25 dB) at frequencies ≥ 1 kHz [10]. However, a major drawback of the preceding HC version was a poor SFP response at frequencies between 125 and 1000 Hz that fell 13–16 dB below SFP responses reported in literature for an optimal RW coupling [6, 10]. Thus, in the HCv2 the spring design was optimized by finite element simulations to improve low to mid frequency performance of the FMT (see Figure 3(d)). The pronounced S-shape of the spring is more robust to axial deformation and makes the device more compliant (*d* < 200 *μ*m: *K*_HCv1_ = 987 N/m versus *K*_HCv2_ = 93.6 N/m) compared to the previous spring design (Figure 1(c)) which improves the transfer of the FMT vibration to the RW at low frequencies. Consequently, comparing SFP displacement amplitudes at 0.125–1 kHz, the new optimized HCv2 provided up to 25 dB higher SFP responses in RW stimulation.

SFP responses to HCv2/FMT stimulation showed a frequency specific dependence on the applied static RW load (Figure 3(b)). Maximal SFP displacement amplitudes between 125 and 1000 Hz were observed at RW loads < 20 mN while SFP responses at higher frequencies reached their maximum at higher forces (≥40 mN). Here two opposite effects can be identified. When a tight connection between HC and RWM is established the mass load of the system is increased by the mass of the cochlear fluids, resulting in an upshift of the resonance frequency of the system (Figure 3(a)). On the other hand, transfer function magnitudes from HC-tip to SFP (Figure 4(a)) were relatively flat across frequency and increased with RW prestress indicating an improved hydraulic ratio from the HCv2 tip to the SFP. This even counteracts the reduction of SFP displacement response seen in Figure 3(a) at frequencies < 1 kHz that was caused by a decrease in FMT output amplitude. Our interpretation is that this is due to the increased nonlinear stiffness of the RWM (Figure 1(c)), leading to a reduced output of the mass-based FMT that provides limited force at low frequencies.

In order to define an optimal RW preload SFP responses to RW stimulation were averaged at speech relevant frequencies (0.5–4 kHz) resulting in a maximum at 15 mN static RW load (Figure 3(c)). The maximum of the average SFP response occurred at a higher RW load when compared to results with the previous HC (4–6 mN). This can be attributed to the more compliant, optimized HCv2 spring design causing less damping on FMT displacement amplitudes at frequencies ≤ 1 kHz even at increased RW loads. The optimal RW load of 15 mN is also in accordance with a study from Gostian et al. [16]. Here the RWM was loaded by a FMT mounted on a translation stage and highest SFP responses to RW stimulation were observed at static forces < 20 mN.

SFP displacement amplitudes in response to HCv2 stimulation at 15 mN static RW preload were compared to SFP responses reported in a study from Arnold et al. [6]. In their optimal and best performing configuration (FMT with underlying fascia) SFP responses were slightly higher (Δ ≤ 3 dB) than responses achieved with the HCv2 at frequencies 0.5–8 kHz except at 2 kHz where SFP displacements with the HCv2 were ~8 dB higher.

Interexperimental min–max ranges in SFP responses to HCv2 stimulation amounted to ~20 dB. Between 0.5–2 kHz they were up to 22 dB lower than the range reported in Arnold et al. [6]. Between 3 and 7 kHz min–max ranges of the HCv2 were similar (Δ ≤ 5 dB) to the range reported in this study (Figure 3(d)). Thus, RW stimulation with the HCv2 led to improved or equal interindividual variability in SFP responses compared to a previous study on optimal FMT-RW coupling with underlying fascia.

Total harmonic distortions of the FMT and SFP displacement amplitudes at frequencies 1, 1.5, and 2 kHz showed no clear dependence on the applied RW load and were generally low (≤1%). Only for the 500 Hz response a strong increase with force was observed, but average SFP response remained below 3% for forces ≤ 80 mN. As described in previous works of our group [10] high THDs at 500 Hz were most likely caused by the ~1.5 kHz resonance in FMT vibrations that resulted in a pronounced 2nd harmonic. However, driving the FMT under highest RW loads is not desirable considering safety and RWM integrity. For an RW load of 15 mN median THDs were low (≤1%).

Taking the considerations of maximum SFP displacement amplitudes, low THDs and safety considerations, that is, avoiding possible RW ruptures, into account, the authors suggest an optimal static RW preload with the HCv2 of ~15 mN. Since estimating such force can be challenging for surgeons with no access to force measurements during FMT implantation a visual indicator of the applied static force was incorporated into the design of the device. For this purpose the HCv2 spring has two metal bars at its backside that coincide when a load of 20 mN is reached. The HCv2 which MED-EL is planning to continuatively refer to as the “Round Window Precision Coupler” requires future testing in patient studies to assess and validate its performance in a clinical environment.

## 5. Conclusion

Our experiments demonstrate the improved performance of the novel “Hannover Coupler v2” prosthesis for the FMT compared to the preceding HC prototype. Redesigning the prosthesis with a strong focus on optimizing the spring to gain low frequency (≤1 kHz) performance led to up to 25 dB higher SFP responses in RW stimulation. A static RW load of 15 mN where THDs were low (≤1%) and SFP amplitudes (0.5–4 kHz) were highest was identified as the optimal load. At 15 mN prestress the SFP responses to HCv2 stimulation were similar (Δ ≤ 3 dB) or higher (8 dB at 2 kHz) compared to previously reported results for optimal RW coupling.

## Supplementary Materials

Supplementary Materials Figure 6: SFP displacement amplitudes in response to acoustic stimulation of the tympanic membrane (~94 dB SPL) of ASTM [17] compliant temporal bones (*n* = 10). Black dashed lines represent the ASTM acceptance range and gray dashed lines represent the extended acceptance range [14] used in this study. Figure 7: schematic drawing of the human ear with implanted MED-EL Vibrant Soundbridge when the Floating Mass Transducer (FMT) is placed at the round window of the cochlea.
