# Supplementary material for: Redesign of the Hannover Coupler: Optimized Vibration Transfer from Floating Mass Transducer to Round Window
Source: Biomed Res Int. 2018 May 14;2018:3701954. doi: 10.1155/2018/3701954 (PMC5976918; doi:10.1155/2018/3701954)
Supplement: Supplementary Materials — Figure 6: SFP displacement amplitudes in response to acoustic stimulation of the tympanic membrane (~94 dB SPL) of ASTM [17] compliant temporal bones (n = 10). Black dashed lines represent the ASTM acceptance range and gray dashed lines represent the extended acceptance range [14] used in this study. Figure 7: schematic drawing of the human ear with implanted MED-EL Vibrant Soundbridge when the Floating Mass Transducer (FMT) is placed at the round window of the cochlea. [file 3701954.f1.pdf]

Supplementary information

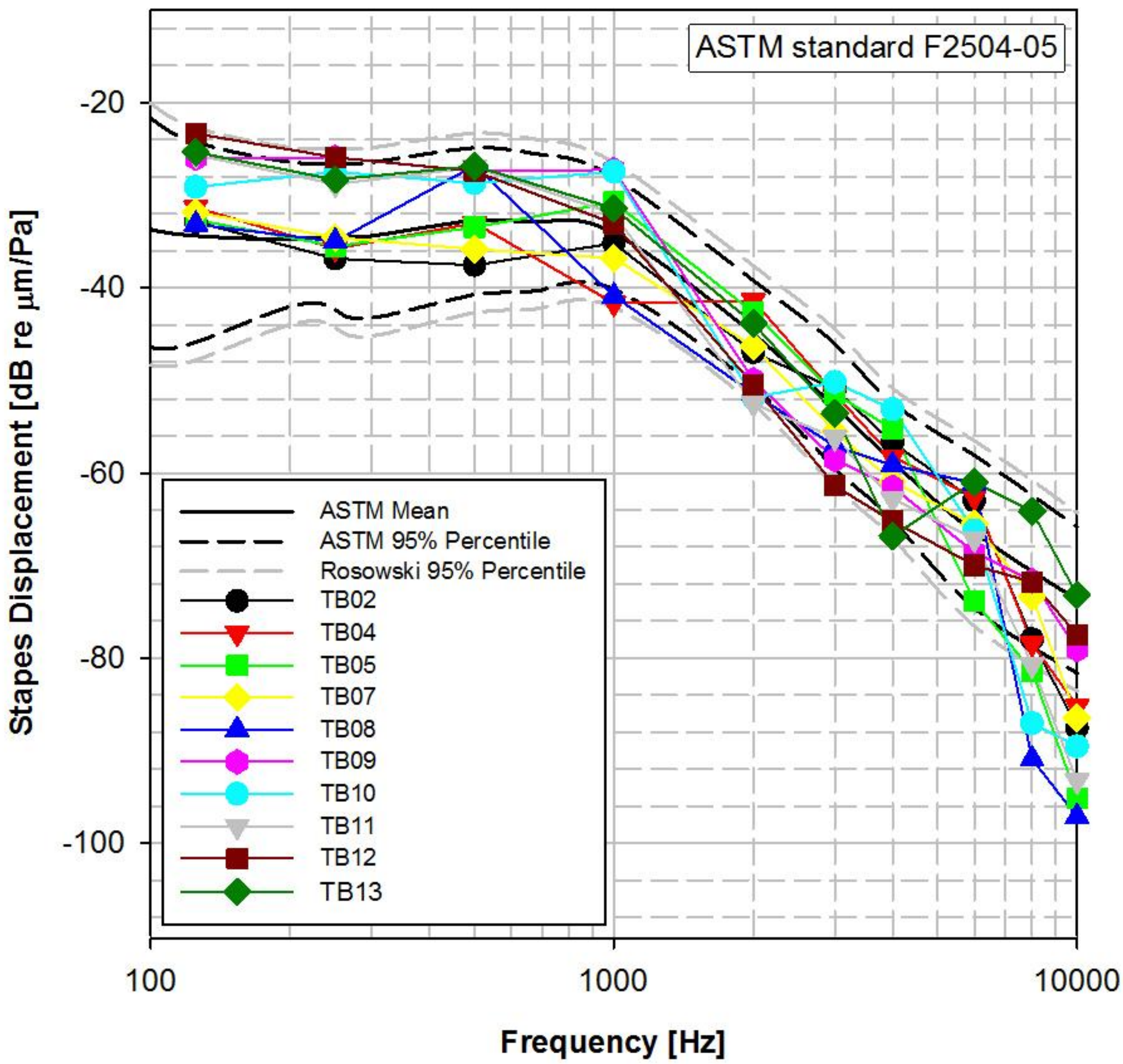

Figure 6: SFP displacement amplitudes in response to acoustic stimulation of the tympanic membrane (~94 dB SPL) of ASTM [17] compliant temporal bones (n=10). Black dashed lines represent the ASTM acceptance range and gray dashed lines represent the extended acceptance range [14] used in this study.

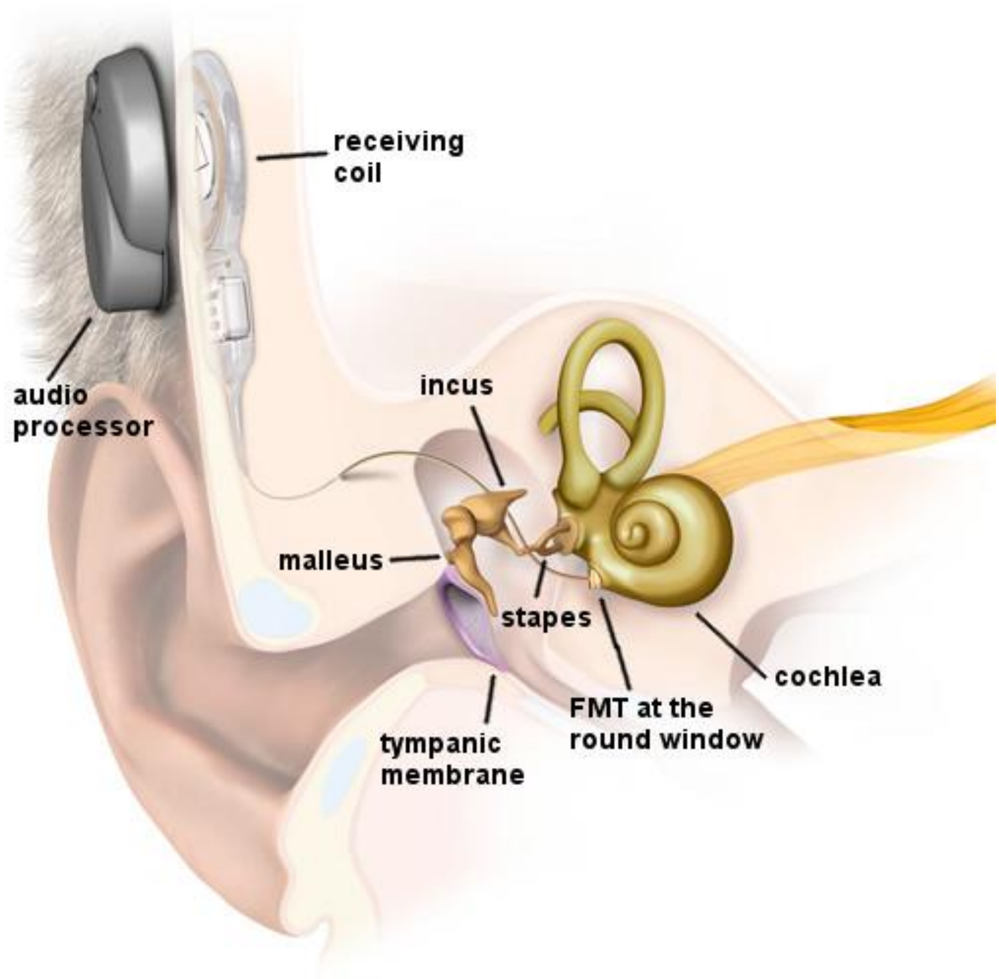

Figure 7: Schematic drawing of the human ear with implanted MED-EL Vibrant Soundbridge® when the Floating Mass Transducer (FMT) is placed at the round window of the cochlea.
